# Nd-Doped ZnO Nanostructures with Enhanced Photocatalytic Performance for Environmental Protection

**DOI:** 10.3390/ijms24076436

**Published:** 2023-03-29

**Authors:** Petronela Pascariu, Corneliu Cojocaru, Petrisor Samoila, Cosmin Romanitan

**Affiliations:** 1 “Petru Poni” Institute of Macromolecular Chemistry, 700487 Iasi, Romania; 2 Department of Chemistry, Institute for Research, Innovation and Technological Transfer of the State Pedagogical University “Ion Creanga”, MD-2069 Chisinau, Moldova; 3 National Institute for Research and Development in Microtechnologies (IMT-Bucharest), 023573 Bucharest, Romania

**Keywords:** electrospinning–calcination method, zinc oxide (ZnO) and Nd (0.05, 0.1, 0.5, 1%)-doped ZnO, photocatalytic activity, methylene blue and ciprofloxacin

## Abstract

Neodymium (Nd)-doped ZnO nanostructures with different amounts of Nd were obtained by the electrospinning–calcination method. X-ray diffraction measurements indicated that the prepared nanostructures have a wurtzite structure without undesirable impurities. Nd doping changes the mean crystallite size as well the lattice strain, as proved by Williamson–Hall plots. The ZnO-based nanostructures were tested as photocatalysts for methylene blue (MB) dye and ciprofloxacin (CIP) drug pollutant degradations under visible light irradiation. Corroborating the obtained results, it was found that the reaction rate constant increased almost linearly with the mean crystallite size (from 2.235 × 10^−2^ to 3.482 × 10^−2^ min^−1^) with a variation in the mean crystallite size from 24.2 to 42.1 nm. Furthermore, the best catalyst sample (0.1% Nd-doped ZnO) was used to optimize the photodegradation process of ciprofloxacin, taking into account the pollutant concentration as well as the catalyst dose. The removal efficiency after 120 min was about 100%, with the rate constant of *k* = 5.291·10^−2^ min^−1^ (CIP) and *k* = 4.780·10^−2^ min^−1^ (MB) for the established optimal conditions. Considering the value of the rate constant, the half-life of the reaction (*τ*_1/2_ = ln2/*k*) was evaluated to be about *τ*_1/2_ =13 min for CIP and 14.5 min corresponding to MB. Several catalytic cycles were successfully performed without any loss of photocatalytic activity using these nanostructures, demonstrating that the obtained nanostructures have good stability in the leaching processes.

## 1. Introduction

Nanostructured oxide semiconductor materials have been intensively studied in recent years due to various commercial applications, including in photocatalysts, batteries, self-cleaning surfaces, fabrics, photovoltaics, antiseptic patches, ink and paints, and so on [1]. Zinc oxide (ZnO) is considered one of the most important n-type semiconductors owing to its outstanding performance and numerous applications (sensors, nanomedicine, optoelectronics, energy, and environment) [2]. It possesses excellent properties such as a wide band gap around 3.34 eV and a high excitation energy of 60 meV, and it preferentially crystallizes in a hexagonal wurtzite-type structure and can form a variety of nanostructures (nanoparticles, nanowires, nanorods, nanofibers, nanospheres, and nanotubes) [3]. Furthermore, due to their large surface area, good compatibility, abundance, nontoxicity, and simple synthesis methods, these nanostructures exhibit unique optical, electronic, physical, and chemical properties [4]. Recently, ZnO has been extensively studied for its photocatalytic performance. Various techniques can be used to prepare this semiconductor, including chemical vapor deposition, molecular beam epitaxy, electrodeposition, electrospinning, sol–gel processes, sputtering, hydrothermal methods, etc. [5,6,7]. On the other hand, electrospinning represents one of the most accessible methods for obtaining these materials due to its low cost and feasibility. The materials prepared by this method have a large active surface, high porosity, and improved functional properties compared to the other techniques [8]. A variety of factors can contribute to improving the performance of ZnO nanostructures as photocatalysts, including their structure, morphology, dopants, band gaps, and structural defects [2]. In this context, rare earth elements are considered ideal dopants to modify the crystal structure, electronic structure, and optical properties of ZnO-based oxide nanostructures, which can effectively influence the positions, widths, and density of the conduction band (CB) and valence band (VB) states [9,10]. Many oxide semiconductor materials based on ZnO doped with various rare earth ions are used for organic pollutant degradation [11,12,13]. Recent developments in pharmaceutical products, such as antibiotics, have presented an environmental and health threat due to their toxicity and nonbiodegradability. Several effects are associated with residues from antibiotics, including the development of antibiotic-resistant microorganisms and the disruption of photosynthetic cycles. A common antibiotic found in wastewater is ciprofloxacin (CIP), a quinolone-type antibacterial used for infections caused by Gram-negative and Gram-positive bacteria [14]. According to our knowledge, there are insufficient studies on the photocatalytic degradation of pharmaceutical products using ZnO catalysts doped with various rare metals under visible light irradiation. In a recent study, Alahmadi et al. [15] used Pt@ZnO nanorods as photocatalysts for ciprofloxacin degradation under visible light irradiation. The authors reported 100% efficiency after 120 min of irradiation.

In our quest to develop new materials with improved photocatalytic performances, Nd-doped ZnO nanostructures were prepared by the electrospinning–calcination method for the degradation of MB dye and CIP drug pollutants. A variety of methods were used in analyzing the photocatalysts, including XRD, SEM, DRS, and fluorescence spectroscopy. The photocatalytic performances of ZnO nanostructures were improved by doping with various amounts (0.05, 0.1, 0.5, 1%) of Nd ions. The photocatalytic performances and kinetic parameters of all materials were assessed using MB (C_0_ = 10 mg/L) as a model organic pollutant. Special attention was dedicated to the synergistic effect of the initial pollutant (ciprofloxacin) concentration (mg/L) and catalyst dosage (%*w*/*v*) on the performance of the photodegradation process in the presence of the best photocatalyst. This material was also evaluated regarding its stability and reusability (after five cycles under the same conditions).

## 2. Results

### 2.1. XRD Analysis

**Figure 1:**
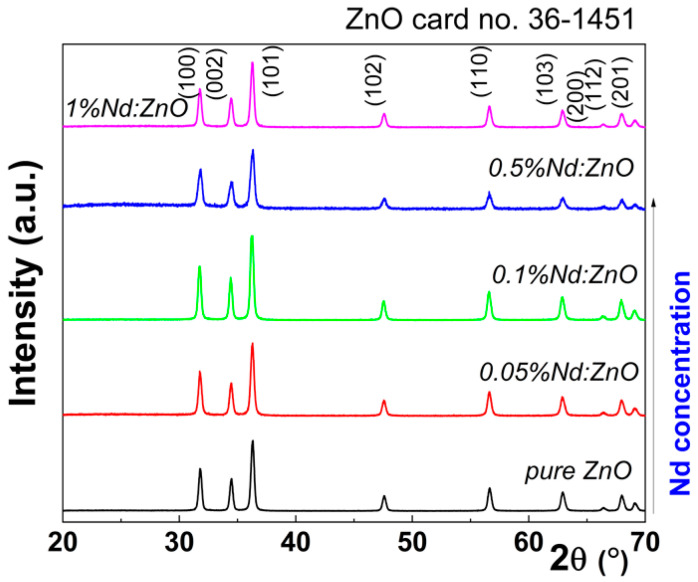
X-ray diffraction patterns for pure ZnO and Nd-doped nanostructures.

The effect of Nd in the crystal structure of ZnO was unveiled using powder X-ray diffraction. Figure 1 shows XRD patterns for the undoped ZnO, as well as at different dopant concentrations.

In each case, XRD patterns present diffraction peaks located at 20 = 31.79, 34.45, 36.26, 47.67, 56.62, 62.89, 66.45, 68.18, and 69.12, further indexed as (100), (002), (101), (102), (110), (103), (200), (112), and (201) reflections of ZnO (ICDD card no. 00-036-1451) with a = b = 0.324 nm and c = 0.517 nm (spatial group P_63_mc(186) with hexagonal symmetry). Other compounds were not identified, confirming the powder purity. Further, the Nd effect on the mean crystallite size and lattice strain was assessed based on size–strain Williamson–Hall (WH) plots. While the band gap value changes slightly after doping with different concentrations of Nd ions for certain systems, the photocatalytic properties are always improved due to intrinsic defect concentration and “impurities” (Nd), since photocatalytic reactions take place in the active sites located on the surface of the nanostructures. The method developed by Williamson and Hall [16] assumes that the diffraction peak has two independent components, namely: size broadening, *β_τ_*, and strain broadening, *β_S_*, and their dependence to the Bragg angle, *θ*, is given by the following relations:

$$\beta_{\tau }=\frac{k\lambda }{\tau cos\theta }$$

(1) $$\beta_{S}=4\varepsilon_{s}tan\theta$$

From Equation (1), one can observe that the size broadening depends on 1⁄cosθ, while the strain broadening depends on *tan*θ. Briefly, the Williamson–Hall method assumes that size and strain contributions are additive factors of the total breadth of the diffraction peak [17,18], thus the convolution is either a simple sum or a sum of squares. Combining Equation (1), it can be obtained:

(2) $$\beta_{tot}=\beta_{\tau }+\beta_{S}=\frac{k\lambda }{\tau cos\theta }+4\varepsilon_{s}tan\theta$$

Then, multiplying this equation by cosθ:

(3) $$\beta_{tot}cos\theta =\frac{k\lambda }{\tau }+4\varepsilon_{s}sin\theta$$

where k is the crystallite shape factor taken as 0.93, τ is the size of the crystalline domains (known as the mean crystallite size), and $\;\varepsilon_{s}$ is the lattice strain.

**Figure 2:**
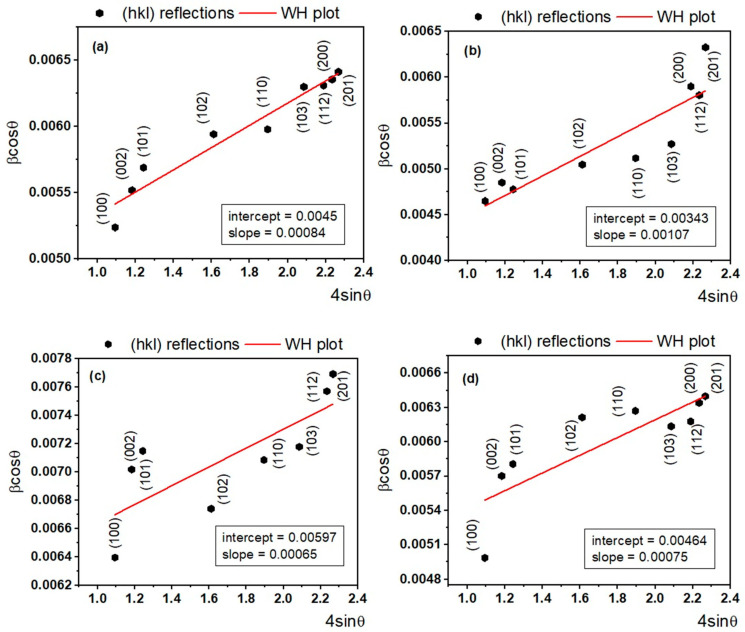
Williamson–Hall plot (red line) on different (hkl) reflections for Nd-doped ZnO samples: (**a**) ZnO:Nd (0.05%), (**b**) ZnO:Nd (0.1%), (**c**) ZnO:Nd (0.5%), and (**d**) ZnO:Nd (1%), respectively.

Figure 2a–d present the Williamson–Hall plots (red line) on multiple reflections for each doped sample with corresponding intercept and slope values as inset, which further provide the value of the mean crystallite size and lattice strain.

Based on the linear plots, the following values for the mean crystallite size and lattice strain were obtained: 31.8 nm (0.05% Nd), 42.1 nm (0.1% Nd), 24.2 nm (0.5% Nd), and 30.8 nm (1% Nd) (mean crystallite size), and 0.084% (0.05% Nd), 0.107% (0.1% Nd), 0.065% (0.5% Nd), and 0.075% (1% Nd) (lattice strain). For the undoped ZnO sample, the value 36.9 nm was obtained.

**Figure 3:**
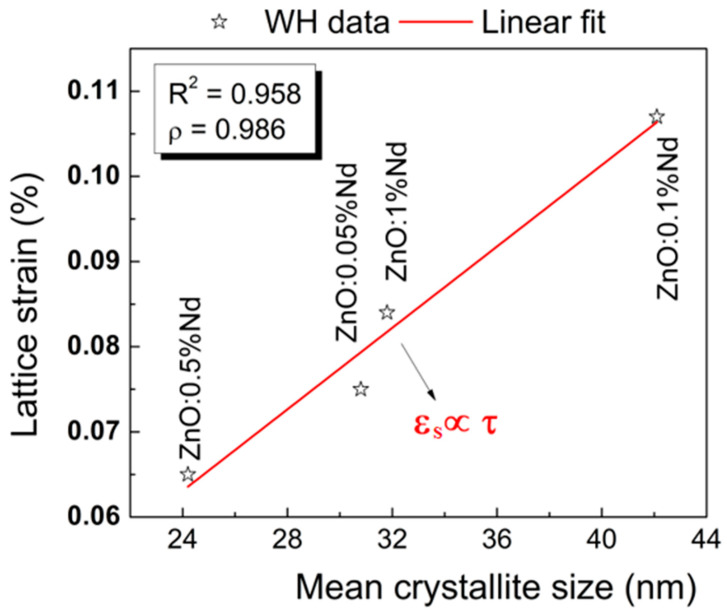
Approximation of the linear fit between lattice strain and mean crystallite size for the doped ZnO samples.

An approximate linear fit was obtained in the dependence between the lattice strain and the mean crystallite size (Figure 3), suggesting that the low crystalline size favors the lattice relaxation, usually ascribed to the formation of structural defects in the lattice. The high correlation between these two microstructural parameters is proved by the ρ Pearson’s correlation parameter, whose value is close to unity. This indicates that the lattice strain and the mean crystallite size are interrelated in our ZnO samples, and later, it is shown that the different reaction rate constants could be explained in this framework. From these results, it can be seen that the concentration of the dopant does not impose a linear behavior of the lattice strain and crystalline size. This aspect is found in many scenarios in the literature [19,20,21], and this nonlinear variation in the lattice strain is due to the variation in the electron affinity of lanthanide ions at a particular concentration. It seems that there is an optimum in terms of the dopant concentration that does not take into account the magnitude of the values for the size of the crystallites and lattice strain.

### 2.2. Morphological Characterization

**Figure 4:**
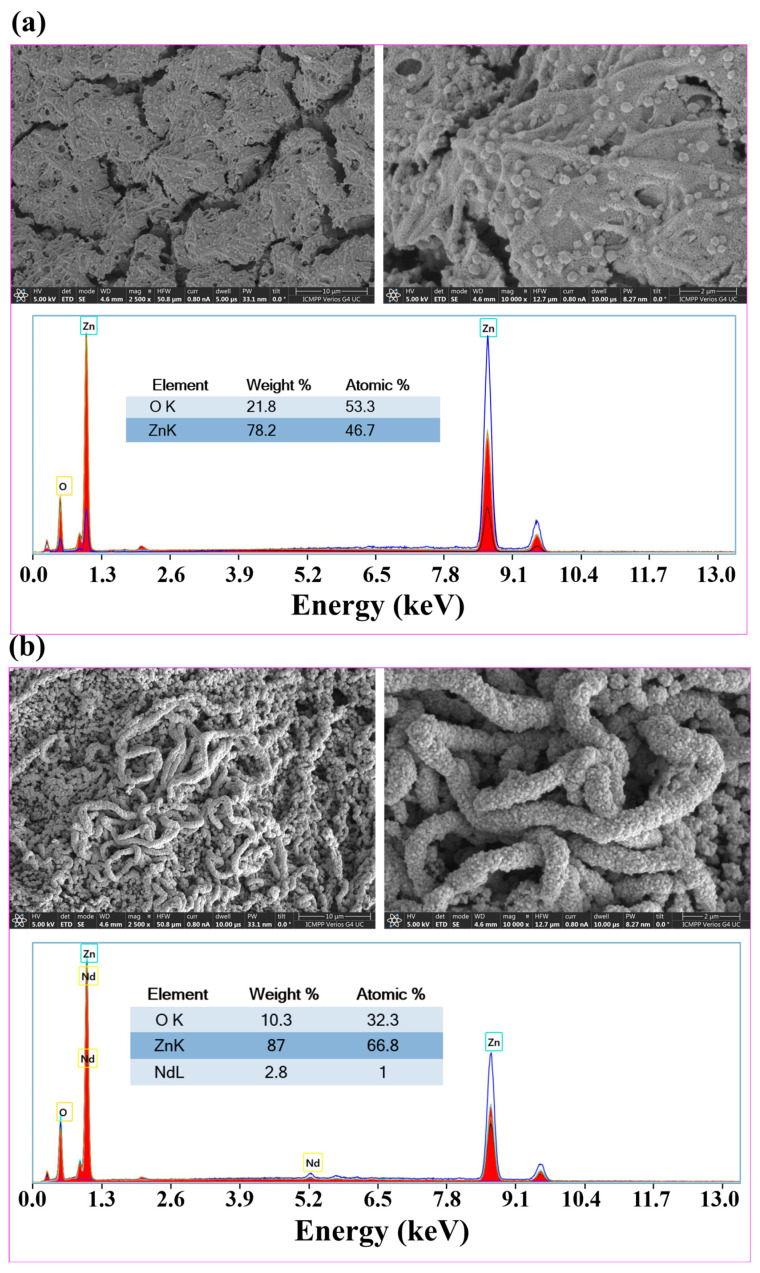
SEM images and EDX spectra of (**a**) undoped ZnO and (**b**) 1% Nd-doped ZnO.

To determine the morphology of pure ZnO and 1% Nd-doped ZnO (e.g., highest dopant amount), SEM micrographs and EDX spectroscopy were employed. Thus, the SEM images of pure (Figure 4a) and 1% Nd-doped ZnO (Figure 4b) nanostructures acquired at different magnifications (×2500 and 10,000) are presented in Figure 4. These materials exhibit characteristic features of nanostructures obtained by the electrospinning–calcination method, represented by one-dimensional worm-like nanofibers composed of interconnected nanocrystals. In addition, the microstructure of the ZnO-based materials obtained by this method keeps its one-dimensional shape, but as expected for this kind of material, the mechanical integrity is quite weak (i.e., the materials are brittle) compared to other semiconductors (for example, TiO_2_) [17]. Energy-dispersive spectrometry (EDX) was used to confirm the presence of Nd ions in the doped materials. Figure 4 shows the EDX spectra of undoped and 1% Nd-doped ZnO samples. The spectra corresponding to the doped material highlighted the presence of Zn, O, and Nd with a 1% value of atomic percentages. We are inclined to believe that the detected difference in the morphology between ZnO and ZnO:Nd (1%) (evidenced by SEM images) might be associated with the fact that these two systems were not equivalent from a thermodynamic standpoint. For the first system (production of ZnO), in the calcination stage, the PVP polymer and acetate anions participated in the burning process. For the second system (production of ZnO:Nd) the burning reaction involved the PVP polymer, acetate anions, and nitrate anions. The latter (nitrate ions) led to the formation of nitrogen dioxide (NO_2_), which is a volatile gas that might contribute to the morphological structuring of the final material during the calcination step.

### 2.3. Optical Analysis

A way to evaluate the photocatalytic properties of oxide semiconductor materials is to perform diffuse reflectance spectroscopy measurements, which enable us to establish the band gap values. The band gap energy (*E*_g_) can be calculated from the UV-Vis reflectance spectra and using the well-known Kubelka–Munk ((Equation (4) and Tauc (Equation (5)) formulas [22,23].

(4) $$F\left(R_{\infty }\right)=\frac{{\left(1\;-\;R_{\infty }\right)}^{2}}{2R_{\infty }}$$

(5) $${\left[F\left(R_{\infty }\right)h\nu \right]}^{\frac{1}{n}}=c{\left(h\nu -E_{g}\right)}^{\frac{1}{n}}$$

where *F*(*R_∞_*) is the Kubelka–Munk function; *R_∞_* represents the absolute reflectance of the materials; *hν* is the photon energy; and *E*_g_ is the band gap energy. Factor *n* can have various values depending on the nature of the electron interband transition, being 1/2 for the direct transition and 2 for the indirect transition band gap, respectively. The *E*_g_ can be evaluated by plotting ${\left[F\left(R_{\infty }\right)h\nu \right]}^{\frac{1}{n}}$ against hν and extrapolating the linear part of the absorption edge to intercept the photon energy axis.

**Figure 5:**
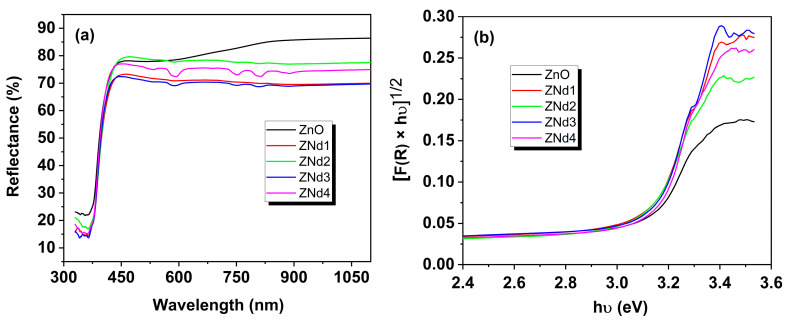

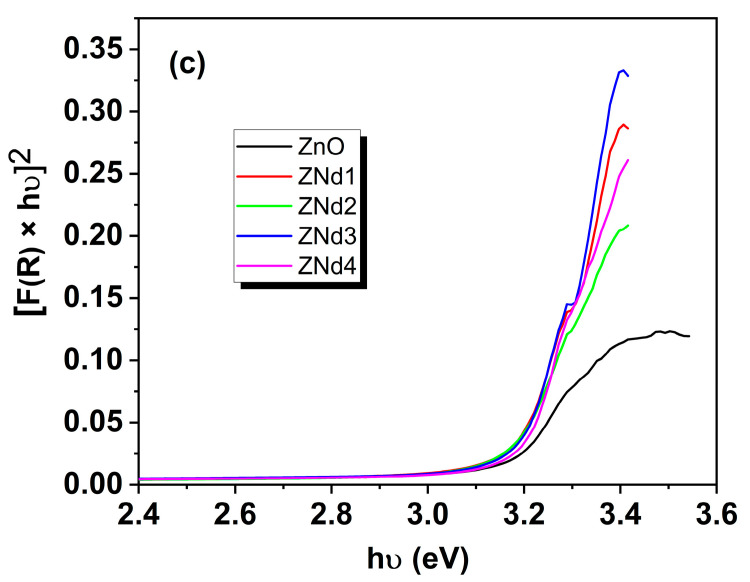
Diffuse reflectance spectra (**a**), ${\left[F\left(R_{\infty }\right)h\nu \right]}^{2}$ (**b**), ${\left[F\left(R_{\infty }\right)h\nu \right]}^{\frac{1}{2}}$ (**c**) plotted against photon energy (hν ) for pure ZnO and Nd-doped ZnO nanostructured materials.

Figure 5a shows the UV-visible diffuse reflectance spectra of pure and Nd-doped ZnO samples recorded between 300 and 1100 nm. To evaluate the *E*_g_ from the Kubelka–Munk calculation, we plotted the graphics using both factor *n* values, 1/2 and 2 (Figure 5b,c, respectively). Table 1 summarizes the obtained values of *E*_g_. As can be seen, the calculated *E*_g_ values are attributed to a factor *n* of 2 (indirect transition band gap). Moreover, there is no important variation between pure and doped ZnO. Many authors have shown that the band gap value changes slightly after doping with different elements for certain systems. For example, Kumar et al. [13] reported slightly higher values after doping with Ce ions (3.32 eV for ZnO and 3.38, 3.36, 3.36 eV for 1, 5, and 10 mol% Ce-doped ZnO materials), which is due to the size reduction in ZnO and the effects of quantum confinement. Another study [24] found higher *E*_g_ values for ZnO doped with Eu compared to ZnO (3.16 eV). Likewise, Ajala et al. [25] showed that an increase in the Al content of ZnO powders results in a slight blue shift in the absorption edge, indicating a slight increase in the bandgap energy (from 3.21 eV for ZnO to 3.23, 3.24, and 3.25 for 0.5, 1.0, and 1.5% for Al-doped ZnO, respectively). Despite the fact that the band gap values reported in all cases do not change significantly, the photocatalytic properties are always improved, as in our case.

### 2.4. Photoluminescence

**Figure 6:**
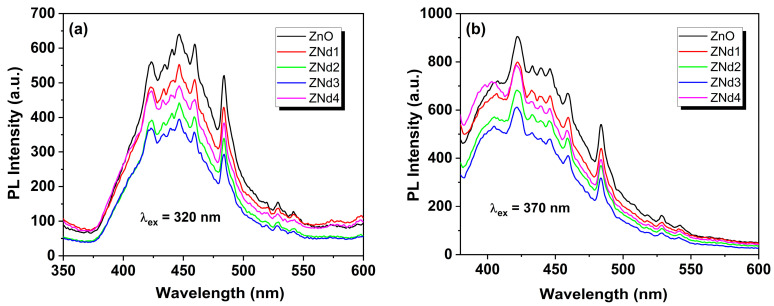
Photoluminescence spectra for pure ZnO and ZnO doped with various amounts of Nd ions (0.05, 0.1, 0.5, 1%) excited at wavelengths of 320 (**a**) and 370 nm (**b**).

The photoluminescence properties of the semiconductor materials can also influence the photocatalytic response. Moreover, semiconductor stoichiometry, intrinsic defect concentration, and impurities can affect the positions and intensity of the band edges in the photoluminescence spectra. Figure 6 shows the emission spectra of pure and Nd-doped ZnO nanostructures excited at wavelengths of 320 and 370 nm, measured between 350 and 600 nm. From these figures, it can be seen that in the visible area of the spectra, there are several bands (423, 434, 440, 446, 459, 484, 528, and 541 nm) due to microstructural defects. Many authors [25,26,27] reported several structural defects in ZnO due to the doping or material preparation method, such as Zn_i_ interstitial zinc, zinc vacancies (V_O_ and V_Zn_), O_i_ oxygen interstitials, and oxygen antisites (O_Zn_). According to Figure 6, the peak positions are similar for all materials, but the intensity of the doped photocatalysts is lower than that of the undoped ZnO. Thus, the decrease in PL intensity in Nd-doped ZnO ascertains that the recombination charge carriers are effectively suppressed by neodymium doping. In consequence, the lower recombination rate of the photogenerated electron–hole pairs increases the lifetimes of electrons and holes, inducing enhanced photocatalytic activity [21].

### 2.5. Photocatalytic Activity

#### 2.5.1. Photodegradation Kinetics under Visible Light Irradiation

In the first part of this study, the screening test was carried out for MB dye degradation with an initial concentration of 10 mg·L^−1^ in the presence of 0.1%*w*/*v* Nd-doped ZnO nanostructures.

**Figure 7:**
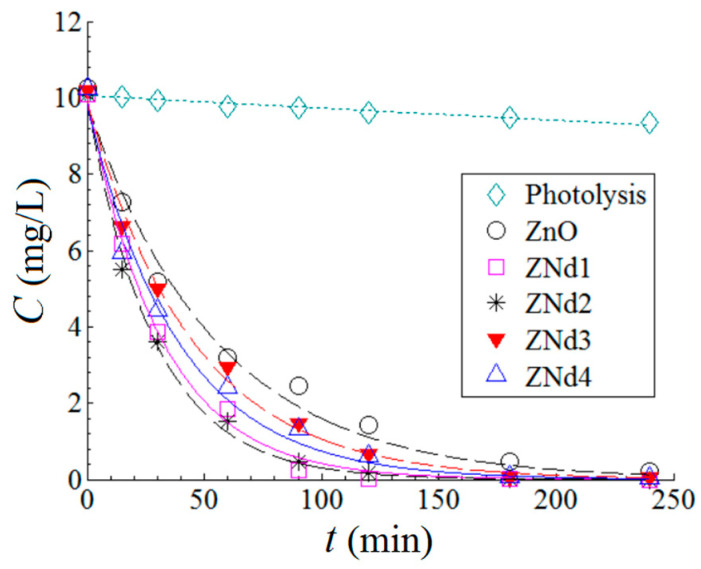
Kinetics of MB dye photodegradation in aqueous solutions under visible light irradiation and in the presence of the produced catalysts (ZnO and ZnO:Nd); solid and dash lines provide predictions according to pseudo-first-order kinetic model; experimental conditions: catalyst dosage = 1 g/L; T = 23 ± 2 °C; pH 7.0 ± 0.2.

Figure 7 displays the photodegradation kinetics of MB dye in aqueous solutions under visible light irradiation and in the presence of the produced catalysts (ZnO and ZnO:Nd). By using the nonlinear regression technique, the experimental data were interpolated to the pseudo-first-order (PFO) kinetic model. According to this PFO model, the time evolutions of the MB dye concentrations can be written as follows:

(6) $$C_{t}=C_{0}\;\;e^{-kt}$$

where *C*_0_—initial MB dye concentration (10 mg/L), *k*—pseudo-first-order reaction rate constant (min^−1^), and *t*—irradiation time (min). The goodness-of-fit was assessed by the error function (*ε^2^*) representing the sum of squares of residual errors (*ε^2^=Σ(C(t)_exp_ − C(t)_calc_)^2^*). The smaller the error function (*ε^2^*), the better the prediction of the PFO model. The fitted parameters of the PFO model are reported in Table 2.

According to Table 2, the amount of the doping element (Nd) in the ZnO matrix of the catalyst has a significant influence. Moreover, the optimal amount of the doping element (Nd) was found to be 0.1% *w*/*w*. Thus, the optimal formulation of the catalyst was attributed to the ZNd2 sample (ZnO:Nd (0.1%)), which disclosed the maximum value for the constant (*k* = 3.482 × 10^−2^ min^−1^). Furthermore, reaction rate constant decreased to 3.174 × 10^−2^ min^−1^ (ZnO:Nd (0.05%), then to 2.579 × 10^−2^ min^−1^ (ZnO:Nd (1%)), reaching 2.235 × 10^−2^ min^−1^ for the ZnO:Nd (0.5%) sample. An explanation of the different observed reaction rate constants could be provided considering the results related to the doped ZnO microstructures or their optical properties. While the bandgap was almost constant at different Nd doping percents, the mean crystallite size was significantly affected. Previous reports showed that the increase in the mean crystallite size enhances the photodegradation of phenol using anatase nanoparticles [28], the photodecomposition rate of organic material using WO_3_ particles [29], or the photocatalytic degradation of phenol of ZnO nanoparticles [30]. In our case, corroborating the results of the photocatalytic activity with the XRD findings, one can observe that the reaction rate constant, k, increased as the mean crystallite size increased. Moreover, considering the linear fit between mean crystallite size and the lattice strain, one can infer that the reaction rate constant decreases in the presence of the lattice relaxation (at small lattice strain).

#### 2.5.2. Design of Experiments (DoE) for Empirical Modeling and Optimization of the Process

**Figure 8:**
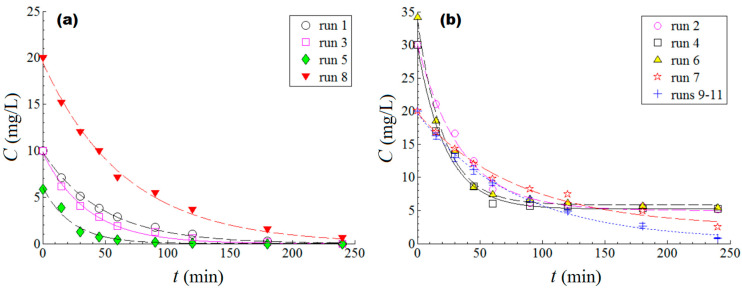
Kinetics of ciprofloxacin photodegradation in aqueous solutions measured for each run (experimental condition) performed under visible light irradiation and in the presence of the produced catalyst (ZnO:Nd (0.1%)): (**a**) experimental data fitted to pseudo-first-order (PFO) kinetic model; (**b**) experimental data fitted to pseudo-first-order with stable component (PFO-SC) kinetic model.

The photodegradation process of organic pollutants in the presence of metal oxide catalysts is influenced by various parameters such as the light intensity, the initial concentration of the pollutant, the type and mass of the photocatalyst, the morphology, shape, and surface of the photocatalyst, the temperature of the solution, the pH, and the irradiation time. Photocatalyst dosage depends on each system and is essential for the degradation process, but the most common catalyst loading range is between 0.04 and 5.0 g/L [31]. It is known that the efficiency of the photodegradation increases with the increase in catalyst loading due to the increase in active sites in contact with the pollutant. However, there is a limit up to which the amount of catalyst has maximum efficiency, after which it decreases due to several factors (increased turbidity of the solution leads to a decrease in light transmission, reduction in the surface area available for the light to promote the generation of h^+^/e^−^ pairs due to particle agglomeration, and surface deactivation caused by particle collisions). This section deals with evaluating the synergetic effect of two important factors, initial pollutant concentration *C_0_* (mg/L) and catalyst dose (*CatDose*,%*w/v*), on the performance of the photodegradation process. To this end, the design of experiments (DoE) and response surface methodology (RSM) were used as the main chemometric tools. One may find more details regarding the mentioned methodologies (DoE and RSM) in the literature [30,31]. In these experiments, ciprofloxacin (CIP) was assayed as an organic pollutant dissolved in water. The experiments were conducted at room temperature, 23 ± 2 °C, and at a naturally occurring pH (7.0 ± 0.3). The performance of the photodegradation process was expressed through the removal efficiency *Y* (response of the process) that was recorded after an irradiation time of 120 min. Hence, the objective of the optimization implied the maximization of the removal efficiency $Y\left(\%\right)$, which can be written as:

(7) $$Y=\left(1-\frac{C_{t}}{C_{0}}\right)\times 100$$

where $C_{0}$ denotes the initial concentration of the CIP organic pollutant and $C_{t}$ is the residual concentration of the organic pollutant (CIP) recorded after *t* = 120 min irradiation time in the course of the photodegradation process. According to modeling methodology, both factors ($C_{0}$ and *CatDose*) were converted into coded dimensionless variables *x_1_* and *x_2_*. This converting operation was performed to compare the effect of factors in the same dimensionless scale. The mathematical equations used for converting actual factors into coded variables are provided elsewhere [32,33]. To explore the photodegradation process in a systematic way, a central composite design of rotatable type (DoE) was adopted for experimentation (Table 3). As highlighted in Table 3, the factors are reported as actual values ($C_{0}$ and *CatDose*) as well as coded levels (*x_1_* and *x_2_*). The experimental plan provided in Table 3 relied on 11 experimental runs, where both factors ($C_{0}$ and *CatDose*) were varied simultaneously. As a result, the process response (Y%) was determined for each run (set of conditions). The central runs (numbers 9 to 11) were performed to estimate the reproducibility of the experiment. Note that for each run (shown in Table 3), the kinetics profile of the ciprofloxacin photodegradation was recorded, as shown in Figure 8. Experimental data were fitted (by using a nonlinear regression technique) to pseudo-first-order (PFO) and pseudo-first-order with stable component (PFO-SC) kinetic models [34]. The determined values for the rate constant are reported in Table S1 (in the Supplemental Materials). For the studied system (CIP/ZnO:Nd (0.1%)), the rate constant varied from 1.230·10^−2^ min^−1^ to 4.743·10^−2^ min^−1^ (see Table S1).

Based on the experimental matrix (Table 3), an empirical mathematical model was constructed using the multiple regression method [32,34]. Hence, the developed empirical model (in terms of coded variables *x_1_* and *x_2_*) can be written as given:

(8) $$\hat{Y}=74.71-5.87x_{1}+4.01x_{2}-0.70x_{1}x_{2}-9.23x_{1}^{2}\;\mathrm{subjected}\;\mathrm{to}:-1.414\leq \;x_{j}\;\leq +1.414;\;j=1,\;2$$

The resulting empirical model (Equation (8)) was validated from the statistical viewpoint by the analysis of variance (ANOVA) [35]. The statistical estimators provided by ANOVA are listed in Table 4.

As reported in the ANOVA table (Table 4), the *F-value* of 14.05 and a small *p*-value (0.0033) suggested a significant model from a statistical standpoint. Therefore, the model can be employed for the predictions in the region of experimentation (valid region). The value of the determination coefficient *R*^2^ revealed that the empirical model might explain about 90% of data variation. Moreover, the adjusted coefficient *R*^2^_adj_ was somewhat smaller than *R*^2^, disclosing that the data-driven model provided satisfactory estimations. The empirical mathematical model in terms of actual factors was detailed using the substitution technique, and it is given by:

(9) $$\hat{Y}=107.10-4.07C_{0}+108.37\times CatDose-1.41\times C_{0}\times CatDose+0.09\times C_{0}^{2}\;\mathrm{subjected}\;\mathrm{to}:\;\;\;\;5.9\;\leq C_{0}\leq 34.1\;\left(\frac{mg}{L}\right);\;\;\;\;\;\;\;\;\;\;\;0.08\leq CatDose\leq 0.22\;\left(\%\frac{w}{v}\right)$$

**Figure 9:**
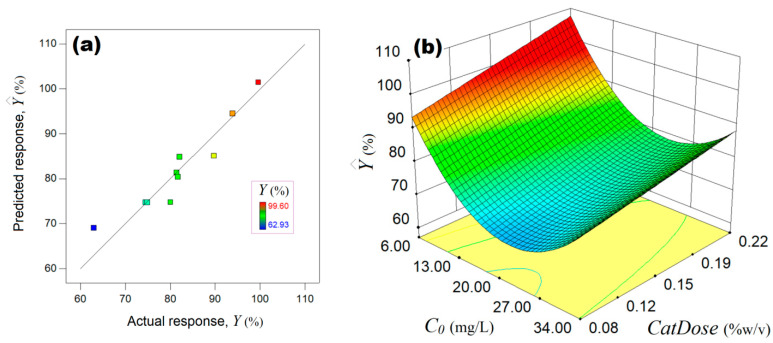
Outcomes of empirical modeling by RSM: (**a**) agreement between experimental observations and model estimations; (**b**) response surface plot predicted by the empirical model showing the synergistic influence of $C_{0}$ and *CatDose* factors on the estimated response ($\hat{Y}$,%).

Figure 9 shows the outcomes of empirical modeling by RSM. The accordance between the experimental data (actual response) and model estimations is highlighted in Figure 9a. Herein, scattering the data around the bisector (45° straight line) indicates a good agreement between the experiment and mathematical model (Figure 9a).

The final empirical model in terms of actual factors (Equation (9)) was used to plot the response surface diagram and highlight the synergetic effect of factors $C_{0}$ and *CatDose* on the process performance (Figure 9b). According to Figure 9b, the main effect of the initial concentration $C_{0}$ is negative, whereas the main effect of the *CatDose* factor is positive with respect to the estimated process response ($\hat{Y}$,%). This means that the increment of the $C_{0}$ factor participates in the diminishing of the response. Instead, as the *CatDose* factor increases, the estimated response is gradually increased (Figure 9b). The quadratic effect of the *C_0_* factor induced a negative curvature by bending the response surface to a syncline region ($C_{0}$: 16–30 mg/L). Likewise, there is a weak interaction effect between both factors *C_0_* and *CatDose*. According to this, the influence of the *CatDose* factor is somewhat more evident at lower initial pollutant concentrations ($C_{0}$ < 10 mg/L). As one can see from Figure 9b, the reddish zone of the response surface indicates the optimal region, which is located in the following interval of the factors *C_0_* (6–13 mg/L) and *CatDose* (0.19–0.22% *w*/*v*).

**Figure 10:**
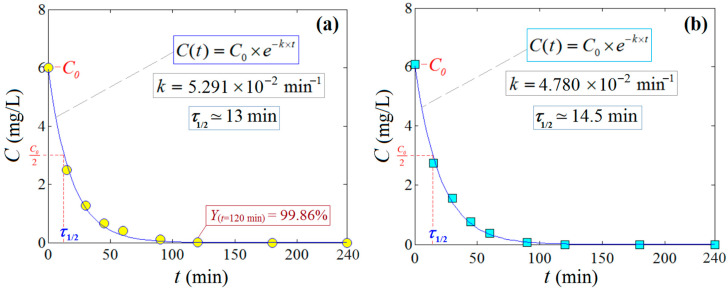
Kinetics profile of photocatalytic degradation (by visible light irradiation) of the CIP pollutant (**a**) and MB dye (**b**) determined under optimal conditions ($C_{0}$ = 6.0 mg/L and *CatDose* = 0.20% *w*/*v*) in the presence of the most efficient catalyst, ZnO:Nd (0.1%).

Ultimately, the developed empirical model was used for the optimization of the investigated photodegradation process. In this sense, the numerical optimization was performed by using the method of simplex (Nelder–Mead variant) [35]. Hence, after model-based optimization, the best possible values of the factors, indicated by the simplex method, were found to be $C_{0}$ = 6.0 mg/L and *CatDose* = 0.20% *w*/*v*. Under these optimal conditions, the calculated response was equal to $\hat{Y}\;$= 106 (predicted value), whereas the observed removal efficiency (recorded at 120 min irradiation time) was found to be Y=99.86% (actual value) for CIP pollutant degradation. The difference of about 6.14% was attributed to the residual error between the model and experiment. This value (99.86%) for the actual response was the maximal one observed in this study. The full kinetics profile for the photodegradation of the CIP pollutant (Figure 10a) and MB dye (Figure 10b) under optimal conditions are illustrated in Figure 10. For these kinetics profiles, the enhanced PFO rate constants (*k*= 5.291·10^−2^ min^−1^) for CIP and (*k*= 4.780·10^−2^ min^−1^) MB were determined. Speaking in terms of the half-life of the reaction (*τ*_1/2_ = ln2/*k*), these values of the rate constants disclosed the half-lives of the reactions of about *τ*_1/2_ =13 min (CIP) and *τ*_1/2_ =14.5 min (MB). Additionally, these results are relevant for the photodegradation process carried out under visible light irradiation. The technical and scientific computations for modeling and optimization were conducted using the Design-Expert 10 and MatLab (v.9.9.0) software programs.

**Figure 11:**
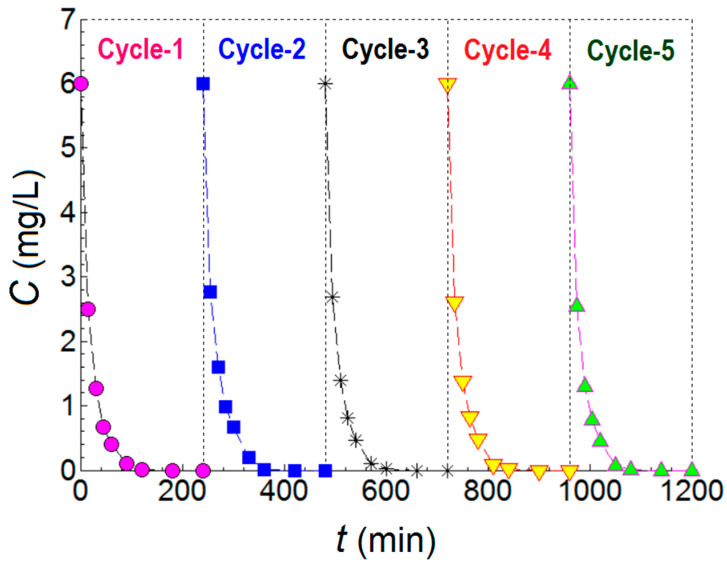
Reuse tests of the ciprofloxacin pollutant degradation in the presence of the ZNd2 sample (ZnO:Nd (0.1%)) for the five evaluations under optimal conditions.

Additionally, we found that ciprofloxacin was degraded by about 59% under UV light irradiation without a catalyst (photolysis), compared to only 14% in visible light (Figure S1, Supplementary Materials). Therefore, this study pointed out the degradation of both pollutants in mild environmental conditions, involving no acidification of solutions and/or the absence of H_2_O_2_, usually used to boost the photochemical reactions, and using a visible light source. The stability and reusability of the materials with the best photocatalytic performance (ZnO:Nd(0.1%) material) were evaluated for five reuse cycles, according to Figure 11. For all measurements, the experimental conditions and parameters were kept constant. After each use, the photocatalyst was recovered and dried at 80 °C for 12 h and then used for the next cycle. As can be seen from Figure 11, the photocatalytic performances of the material were relevant even after five cycles of use, which confirmed the superior stability of the newly prepared catalyst. Good stability of the ZnO:Nd (0.1%) material was also obtained for the MB degradation (see Figure S2, Supplementary Materials).

A comparative analysis of materials based on ZnO doped with different elements (transition metals and lanthanides) is presented in Table 5. The photocatalytic activities of Nd-doped ZnO are superior to pure ZnO and comparable to or even better than most reported state-of-the-art metal-doped ZnO photocatalysts. According to the data reported in this table, these materials have high degradation efficiencies (~100%) after 120 min of visible light irradiation, with reaction rates reaching *k* = 4.780 × 10^−2^ min^−1^ for MB dye degradation and *k* = 5.291 × 10^−2^ min^−1^ corresponding to CIP pollutants. Another advantage of these systems was related to the light conditions, involving no acidification of solutions and/or the absence of H_2_O_2_, usually used to boost the photochemical reactions.

**Figure 12:**
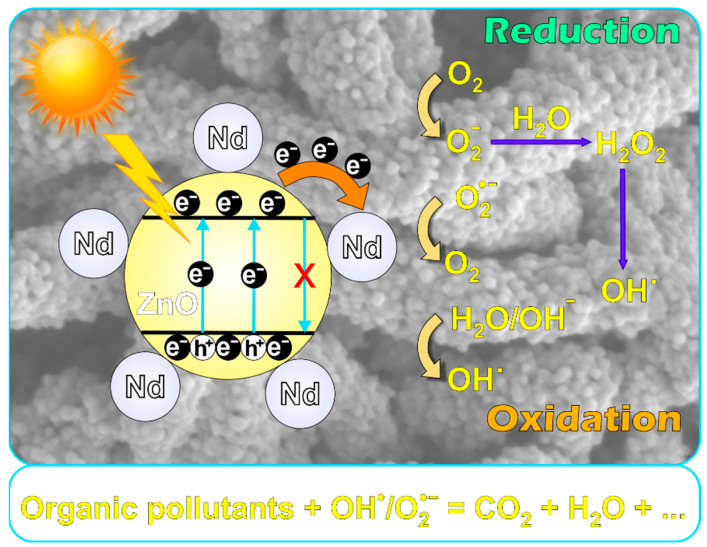
Proposed mechanism of the degradation of organic pollutants in the presence of the Nd-doped ZnO photocatalysts.

According to the available literature [20,41,42,43,44], a proposed mechanism for the degradation of organic pollutants (MB, CIP) in the presence Nd-doped ZnO nanostructures is provided (Figure 12). The light of a certain wavelength is absorbed by the photocatalysts, along with electron excitation from the valence band (VB) to the conduction band (CB). This spawns positive charge carriers (holes, *h^+^*) in the VB of the photocatalysts. The holes in the VB trigger hydroxyl-free radicals ($OH^{•}$) from H_2_O molecules. Simultaneously, the oxygen molecules are converted to oxygen radicals ($O_{2}^{•-}$) due to the capture of the generated electrons (*e^−^*) in the CB. Additionally, secondary reactions can occur, which produce more $OH^{•}$ radicals. The generated $OH^{•}$ radicals trigger the decomposition of the organic pollutants previously adsorbed on the surface of the catalyst resulting in CO_2_, H_2_O, or other byproducts, depending on the pollutant type [41].

## 3. Materials and Methods

### 3.1. Materials

Zinc acetate [Zn(CH_3_COO)_2_·2H_2_O]), neodymium (III) nitrate hexahydrate (Nd(NO_3_)_3_·6H_2_O), N,N-dimethylformamide (DMF), ethanol (EtOH), polyvinylpyrrolidone (PVP) (M_w_ = 1,300,000), methylene blue dye (MB), and ciprofloxacin (CIP, C_6_H_9_N_3_O_3_) were purchased from Sigma-Aldrich (St. Louis, MO, USA) and used without further purification.

### 3.2. Preparation of Nd-Doped ZnO Nanostructures

Pure and Nd (0.05, 0.1, 0.5 and 1%)-doped ZnO nanostructures were obtained by the electrospinning technique, after which they were calcined at 700 °C (15 °C/min heating rate) for 3 h. Undoped ZnO was obtained by dissolving 0.92 g of zinc acetate and 0.72 g of PVP in a solvent mixture of Ethanol:DMF (1:1). The doped materials were obtained by adding different amounts (0.05, 0.1, 0.5, and 1%) of neodymium (III) nitrate to the corresponding solution. After mixing and homogenizing the resulting solution for 12 h, it was transferred to a needle with an inner diameter of 0.5 mm for electrospinning. The composite fibers (PVP + salts) were developed using an electrospinning setup produced by the Fluidnatek^®^ LE-50 laboratory line from Bioinicia S.L. (Valencia, Spain). The main working parameters were set at a high voltage of 25 kV, the distance between the needle tip and the collector of 15 cm, and the feed flow rate of the electrospun solution of 5 µL/min. A square stainless-steel sheet was used to collect the composite fibers. Afterward, the samples were calcined for 3 h in an air atmosphere at 700 °C to completely remove the PVP matrix, and the ZnO and Nd-doped ZnO nanostructures were obtained according to a possible chemical reaction (10) (developed for the case of ZNd4 (1% Nd) as an example). The developed materials were labeled as follows: pure ZnO (ZnO) and Nd (different percentages)-doped ZnO → ZNd1 (0.05% Nd), ZNd2 (0.1% Nd), ZNd3 (0.5% Nd), and ZNd4 (1% Nd).

99Zn(CH_3_CO_2_)_2_ + Nd(NO_3_)_3_ + 395O_2_ → 100Nd_0.01_Zn_0.99_O + 396CO_2_↑ + 297H_2_O↑ + 3NO_2_↑ Anctionte ofanthanides (10)

### 3.3. Characterization of the Prepared Materials

Characterizations of the prepared materials are provided in the Supplementary Materials.

### 3.4. Photocatalytic Tests

Model pollutant photodegradation experiments were conducted using the methylene blue dye (MB) and ciprofloxacin (CIP) drug pollutants in an aqueous solution. A halogen lamp (400 W) was used as a lighting source, with the emission spectrum reported in our previous study [23]. As a first step, screening tests were performed for the degradation of MB (C_0_ = 10 mg/L^−1^) in the presence of pure and Nd-doped ZnO (catalyst dose = 0.1%*w*/*v*) nanostructured materials. The adsorption equilibrium was established by magnetic stirring (500 rotations/min) the catalyst and pollutant mixture in the dark for 30 min before each photodegradation kinetics. According to the established protocol, 3 mL of degradation solution was collected after certain time intervals, then its UV-Vis spectra were recorded using a SPECORD 210^+^ spectrometer (Jena, Germany). The temperature and distance between the light source and the reactor can influence photocatalytic activity. Thus, the experiments were conducted at room temperature with a 30 cm distance between the light source and the reactor. Moreover, the pH value used for all measurements was 7.0 ± 0.3. After selecting the catalyst with the best photocatalytic performance, a protocol was developed to optimize the CIP photodegradation process based on the most important factors (initial pollutant concentration and catalyst dose).

## 4. Conclusions

In this work, pure and Nd-doped ZnO nanostructures obtained by the electrospinning–calcination method were investigated as catalysts for the degradation of MB dye and CIP drug pollutants. The XRD analysis confirmed the crystallinity of the obtained materials with wurtzite symmetry, without identifying any other additional compounds. The investigations showed that the different Nd content changes the mean crystallite size and the lattice strain. The SEM measurements of pure and 1% Nd-doped ZnO nanostructures exhibited one-dimensional worm-like nanofibers composed of interconnected nanocrystals. The photoluminescence properties of the prepared pure and 1% Nd-doped ZnO nanostructures highlighted the presence of microstructural defects that influence the photocatalytic response. Next, the ZnO-based nanostructures were used to photodegrade methylene blue dye and ciprofloxacin drug pollutants from aqueous solutions under visible light irradiation. First, all the prepared materials were tested to find the proper amount of the doping element (Nd) in the ZnO structure to show the best photocatalytic performance. Therefore, the optimal amount of the doping element (Nd) was found to be 0.1% *w*/*w*. For this, the maximum rate constant for MB dye degradation using the Nd (0.1%)-doped ZnO (ZNd2) catalyst was 3.482 · 10^−2^ min^−1^. For this sample, it was also found that the mean crystallite size was the highest. Then, starting from the best photocatalyst, the synergistic effect of two important factors such as the initial pollutant concentration (ciprofloxacin) (mg/L) and the catalyst dose (%*w*/*v*) on the performance of the photodegradation process was evaluated and discussed. Hence, after model-based optimization, the best possible values of the factors, indicated by the simplex method, were found to be $C_{0}$= 6.0 mg/L and *CatDose* = 0.20% *w*/*v*. Under these optimal conditions, the removal efficiencies (recorded at 120 min irradiation time) were found to be Y = 99.86% with the rate constant of *k* = 5.291·10^−2^ min^−1^ for CIP and Y= 100% with the rate constant of *k* = 4.780·10^−2^ min^−1^ corresponding to MB dye degradation. Regarding the half-life of the reaction (*τ*_1/2_ = ln2/*k*), the value of the rate constant disclosed a reaction half-life of about *τ*_1/2_ = 13 min (for CIP) and *τ*_1/2_ = 14.5 min (for MB). Likewise, the optimized material showed excellent stability in the recovery processes without any decrease in their photocatalytic performance after five reuse cycles.

## Figures and Tables

**Table 1:** Optical band gap (*E*_g_, eV) values of the prepared materials.

Samples	*E*_g_ (eV)	
	*n* = ½	*n* = 2
ZnO	3.03	3.13
ZnO:Nd (0.05%) → ZNd1	3.05	3.16
ZnO:Nd(0.1%) → ZNd2	3.01	3.14
ZnO:Nd(0.5%) → ZNd3	3.05	3.18
ZnO:Nd(1.0%) → ZNd4	3.08	3.16

**Table 2:** Kinetic parameters for MB dye photodegradation under visible light in the presence of the produced catalysts (ZnO and ZnO:Nd).

Material Code	Material Formulation	Pseudo-First-Order of the Reaction Rate Constant, *k* (min^−1^)	Error Function (*ε*^2^)
--	--/Photolysis	3.339 × 10^−4^	0.028
ZnO	ZnO	1.817 × 10^−2^	0.907
ZNd1	ZnO:Nd (0.05%)	3.174 × 10^−2^	0.290
ZNd2	ZnO:Nd (0.1%)	3.482 × 10^−2^	0.272
ZNd3	ZnO:Nd (0.5%)	2.235 × 10^−2^	0.432
ZNd4	ZnO:Nd (1.0%)	2.579 × 10^−2^	0.977

**Table 3:** Central composite design (rotatable type) used for experimentation of ciprofloxacin photodegradation in the presence of ZnO:Nd (0.1%) catalyst.

Run	Initial Concentration of Pollutant (mg/L)		Catalyst Dose (% *w*/*v*)		Removal Efficiency (Response), Recorded After 120 min Irradiation Time
	*Coded*	*Actual*	*Coded*	*Actual*	
	*x_1_*	*C_0_*, mg/L	*x_2_*	*CatDose*, % *w/v*	*Y* (%)
1	−1	10.0	−1	0.10	89.70
2	+1	30.0	−1	0.10	80.03
3	−1	10.0	+1	0.20	93.91
4	+1	30.0	+1	0.20	81.43
5	−1.414	5.9	0	0.15	99.60
6	+1.414	34.1	0	0.15	82.08
7	0	20.0	−1.414	0.08	62.93
8	0	20.0	+1.414	0.22	81.66
9	0	20.0	0	0.15	74.53
10	0	20.0	0	0.15	74.90
11	0	20.0	0	0.15	74.89

**Table 4:** Analysis of variance (ANOVA) for the fitted model $\hat{Y}\;\left(x_{1},\;x_{2}\right)$.

Source	*DF* ^(a)^	*SS* ^(b)^	*MS* ^(c)^	*F-Value* ^(d)^	*p-Value* ^(e)^	*R^2^* ^(f)^	*R_adj_ ^2^* ^(g)^
**Model**	4	932.36	233.09	14.05	0.0033	0.903	0.839
**Residual**	6	99.56	16.59				
**Total**	10	1031.92					

^(a)^ degree of freedom; ^(b)^ sum of squares; ^(c)^ mean square; ^(d)^ ratio between mean squares; ^(e)^ probability of randomness; ^(f)^ coefficient of determination; ^(g)^ adjusted coefficient of determination.

**Table 5:** Comparative data of existing ZnO-based materials in the literature for the degradation of different pollutants.

Catalyst, Dose	Synthesis Method	Pollutant, Initial Concentration	Light Source	*k* (min^−1^)	References
ZnO:Ag (1%) 0.2 g/L	electrospinning–calcination	Amaranth, 11 mg/L	Vis (400 W)	3.229 × 10^−2^	[20]
ZnO:La (2%) 0.2 g/L	electrospinning–calcination	CR, 10 mg/L	UV	2.734 × 10^−2^	[36]
ZnO:Sm (1%) 0.2 g/L	electrospinning–calcination	CR, 10 mg/L	UV	1.337 × 10^−2^	[11]
ZnO:La (2%)/C 0.1 g/L	electrospinning–calcination	RhB [na]	UV	4.270 × 10^−2^	[37]
ZnO:Nd (1%) 0.25 g/L	sol–gel	TC, 15 mg/L	Vis	7.3 × 10^−3^	[38]
ZnO:Nd (1%) 2 g/L	sol–gel	MB, 10 mg/L	UV (125 W)	0.158	[39]
ZnO:La (2%) 5 g/L	solvothermal	RhB 1.0 × 10^−5^ M	UV (15 W)	-	[40]
ZnO:Pt (0.6%) 1.6 g/L	-	CIP	Vis (300 W)	-	[15]
ZnO:Nd (0.1%) 2 g/L	electrospinning–calcination	MB, 6 mg/L	Vis (400 W)	4.780 × 10^−2^	This work
ZnO:Nd (0.1%) 2 g/L	electrospinning–calcination	CIP, 6 mg/L	Vis (400 W)	5.291 × 10^−2^	This work

Note: Type of irradiation of the photocatalytic system: Vis—visible light and UV—ultraviolet light; CR = Congo Red; TC = Tetracicline; CIP = Ciprofloxacin; MB = Methylene Blue; RhB = Rhodamine B.

## Data Availability

Not applicable.

## Supplementary Materials

The following supporting information can be downloaded at: https://www.mdpi.com/article/10.3390/ijms24076436/s1.
