# Supplementary material for: Nd-Doped ZnO Nanostructures with Enhanced Photocatalytic Performance for Environmental Protection
Source: Int J Mol Sci. 2023 Mar 29;24(7):6436. doi: 10.3390/ijms24076436 (PMC10094349; doi:10.3390/ijms24076436)
Supplement: Supplementary file 1 [file ijms-24-06436-s001.zip › ijms-2238481-supplementary.pdf]

Supplementary Material

Nd-doped ZnO nanostructures with enhanced photocatalytic performance for environmental protection

Petronela Pascariu[1*], Corneliu Cojocaru[1,2], Petrisor Samoila[1*], Cosmin Romanitan[3]

[1]"Petru Poni" Institute of Macromolecular Chemistry, 41A Grigore Ghica Voda Alley, 700487, Iasi, Romania

[2]Institute of Research, Innovation and Technological Transfer of the State Pedagogical University "Ion Creanga", Chisinau, MD-2069, Republic of Moldova

[3]National Institute for Research and Development in Microtechnologies (IMT-Bucharest), Bucharest, 023573, Romania

3.3. Characterization of the prepared materials

The crystalline structures of nanomaterials were analyzed using an 9kW Rigaku SmartLab XRD diffractometer- (Rigaku Corporation, Japan). The powder XRD patterns were recorded in 2θ mode with step of 0.01° and speed of 12°/min. SEM micrographs were obtained using Verios G4 UC Scanning Electron Microscope (Thermo Fisher Scientific) equipped with an energy dispersive spectrometer (EDS, EDAX Octane Elite). Diffuse reflectance spectra (DRS) were recorded by Analytik Jena SPECORD UV–Vis 210+ spectrophotometer, equipped with an integrating sphere accessory, in a wavelength range of 200-1100 nm. Photoluminescence spectra were assessed using a Perkin Elmer LS55 (PerkinElmer) spectrophotometer.

[1*] Corresponding author: dorneanu.petronela@icmpp.ro; pascariu_petronela@yahoo.com (Petronela Pascariu); samoila.petrisor@icmpp.ro (Petrisor Samoila)

Table S1. Kinetic rate constants determined for each run of DoE.

Run	Fitted Model	Rate constant, k (min^{-1})	Error function, ε^2
1	PFO *)	2.072×10^{-2} min^{-1}	0.178
2	PFO-SC **)	2.875×10^{-2} min^{-1}	3.886
3	PFO	2.765×10^{-2} min^{-1}	0.454
4	PFO-SC	4.346×10^{-2} min^{-1}	4.851
5	PFO	4.225×10^{-2} min^{-1}	0.684
6	PFO-SC	4.743×10^{-2} min^{-1}	4.150
7	PFO-SC	1.242×10^{-2} min^{-1}	3.167
8	PFO	1.517×10^{-2} min^{-1}	1.614
9	PFO-SC	1.241×10^{-2} min^{-1}	1.474
10	PFO-SC	1.401×10^{-2} min^{-1}	1.293
11	PFO-SC	1.230×10^{-2} min^{-1}	0.984

*) PFO kinetic model: $C(t)=C_0 \times \exp(-kt)$

**) PFO-SC kinetic model: $C(t)=(C_0 - C_s) \times \exp(-kt) + C_s$, where C_s is the portion of organic pollutant which is stable.

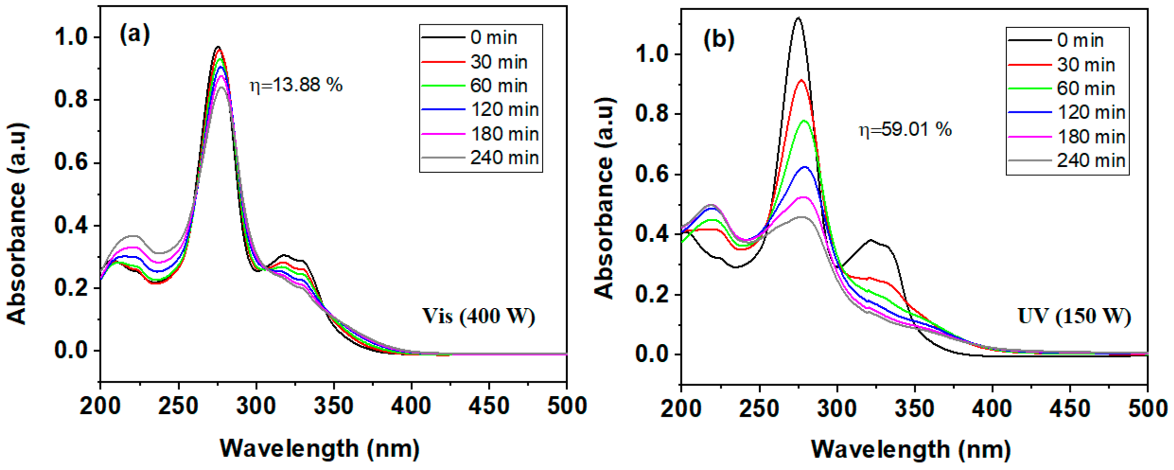

Fig. S1. UV-VIS absorption spectra of the photolysis test for Ciprofloxacin pollutant performed under visible (a) and UV (b) light irradiation for 240 minutes.

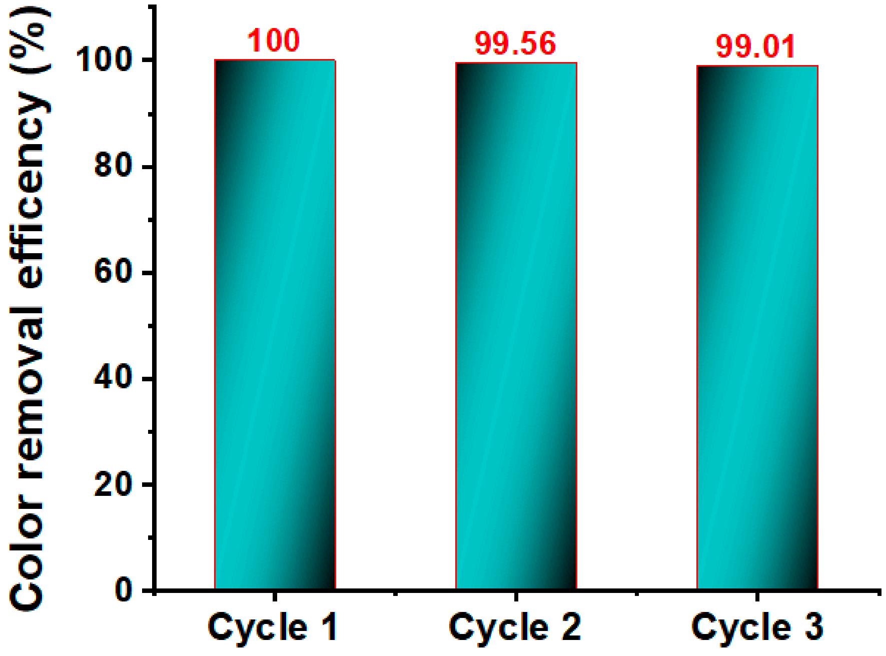

Fig. S2. Reuse of ZnO:Nd(0.1%) material for three cycles to remove MB after 120 min of visible light irradiation.
